# A High-Throughput Oxidative Stress Biosensor Based on *Escherichia coli* roGFP2 Cells Immobilized in a *k*-Carrageenan Matrix

**DOI:** 10.3390/s150202354

**Published:** 2015-01-22

**Authors:** Lia Ooi, Lee Yook Heng, Izumi C. Mori

**Affiliations:** 1 Southeast Asia Disaster Prevention Research Initiative (SEADPRI-UKM), Institute for Environment and Development (LESTARI), National University of Malaysia, 43600 Bangi, Selangor, Malaysia; E-Mails: angelyn.ooi.lia@gmail.com (L.O.); leeyookheng@yahoo.co.uk (L.Y.H.); 2 Faculty of Science and Technology, National University of Malaysia, 43600 Bangi, Selangor, Malaysia; 3 Institute of Plant Science and Resources, Okayama University, Kurashiki 710-0046, Japan

**Keywords:** oxidative stress biosensor, roGFP2, immobilized whole-cell biosensor, redox-sensitive GFP, arsenite, selenite, metalloids, ROS

## Abstract

Biosensors fabricated with whole-cell bacteria appear to be suitable for detecting bioavailability and toxicity effects of the chemical(s) of concern, but they are usually reported to have drawbacks like long response times (ranging from hours to days), narrow dynamic range and instability during long term storage. Our aim is to fabricate a sensitive whole-cell oxidative stress biosensor which has improved properties that address the mentioned weaknesses. In this paper, we report a novel high-throughput whole-cell biosensor fabricated by immobilizing roGFP2 expressing *Escherichia coli* cells in a *k*-carrageenan matrix, for the detection of oxidative stress challenged by metalloid compounds. The *E. coli* roGFP2 oxidative stress biosensor shows high sensitivity towards arsenite and selenite, with wide linear range and low detection limit (arsenite: 1.0 × 10^−3^–1.0 × 10^1^ mg·L^−1^, LOD: 2.0 × 10^−4^ mg·L^−1^; selenite: 1.0 × 10^−5^–1.0 × 10^2^ mg·L^−1^, LOD: 5.8 × 10^−6^ mg·L^−1^), short response times (0–9 min), high stability and reproducibility. This research is expected to provide a new direction in performing high-throughput environmental toxicity screening with living bacterial cells which is capable of measuring the bioavailability and toxicity of environmental stressors in a friction of a second.

## 1. Introduction

Aquatic ecosystems can be disrupted by chemical pollution. Many environmental pollutants can alter the intracellular oxidation status of an organism via oxidative damage which causes biochemical dysfunction and ultimately may lead to cell death. Oxidative stress occurs as a result of an imbalance in cellular redox status, such as a rapid production of reactive oxygen species (ROS) which exceeds the capacity of the antioxidant defense mechanisms of the cells [1]. Metalloid oxyanions are among the environmental stressors which cause toxicity in organisms via alteration of cellular redox status [2]. Metalloids are elements intermediate in properties between the typical metals and non-metals. Among the reported environmental stressors, arsenite and selenite are two relevant metalloids, which are known to induce oxidative stress in cells leading to cytotoxicity [3–5]. Conventionally, changes in redox balance of cells were examined by determination of glutathione/glutathione disulfide (GSH/GSSG) ratio, which involves complicated, destructive sample preparation steps and relatively long processing times [6]. Therefore, the development of quick, simple, non-destructive biosensors using living cells which allow the detection of chemical-induced cellular oxidation stress should be very beneficial for risk management of aquatic ecosystems.

Green fluorescent protein (GFP) was first found in *Aequorea victoria* 53 years ago [7]. It is made up of 238 amino acids in a single polypeptide chain, which gives out green fluorescence when irradiated with far-UV light [8]. Due to the fundamental advantage of GFP to generate fluorescence with great stability in live tissues and cells, GFP provides great benefit and flexibility in evaluating reporter activity [9,10]. GFP has been used as a reporter gene, fusion tag, cell marker, pH indicator and biosensor for organellar, cellular and environmental applications [9,11]. Hazardous chemicals in the environment can result in stress formation in living cells, which changes the redox equilibrium of the cell, thus influencing the differentiation, communication, gene transcription, immune response, growth, stress responses, metabolism, migration, ion channels, cell cycle *etc.*, of cells [12–14]. Excess oxidation which overwhelms the reductive abilities of cell leads to death. Many redox-sensitive GFP (roGFP) variations have been created to monitor redox status of cells [15,16].

roGFP2, which possesses two cysteine residues at the positions 147 and 204, has been found to be more suitable with its high intensity of fluorescence and long dynamic range compared with other roGFP variations. The 4-hydroxybenzylidene-imidazolinone chromophore, which is also known as Y66 chromophore, is the chromophore in roGFP2, that emits fluorescence [17]. The changes in cell redox potential (E_GSH_) enhance the formation of disulfide bonding between cysteine residues which triggers protonation of the roGFP2 Y66 chromophore, leading to an excitation peak shift [15,18,19]. The ratio of fluorescence intensity at the 400 and 490 nm excitation peaks of the protein can be referred to determine the oxidation level and redox potential enhanced by a particular chemical. Determination of oxidation level via calculation of 400/490 nm ratio eliminates cell quantity factor and variables factors such as cell thickness, instrumental sensitivity, light intensity and photobleaching, it also differentiates actual redox changes from artifacts such as arylation, meanwhile it removes errors caused by variations in roGFP2 concentration during different growth phases in *E. coli* roGFP2 cells [12,19]. The authors of [3] reported a sensitive oxidative stress biosensor using *E. coli* roGFP2 cells in buffer suspension. The biosensor was however found to be unstable for long-term storage (∼10 h, Arias-Barreiro and Mori, unpublished data). We overcame this weakness by immobilizing the bacterial cells in a non-toxic matrix. The aim of this study was to fabricate a sensitive oxidative stress biosensor which is easy to produce, and has high-throughput and with long term stability. In this paper, we report a sensitive oxidative stress biosensor fabricated from roGFP2-expressing *E. coli* immobilized in *k*-carrageenan (KC).

To compare the characteristics of the KC-entrapped *E. coli* roGFP2 biosensor to other previous toxicity biosensors, we selected arsenite and selenite as model environmental stressors. Arsenic that is found in the environment occurs from both natural and anthropogenic sources. Naturally occurring arsenic-containing bedrock formations are highly related to well water and ground water pollution episodes in Bangladesh, West Bengal (India) and regions of China [5,20]. Human activities, particularly gold mining (e.g., in Ghana, UK and Thailand), coal burning (in Slovakia, Turkey and China) and the use of arsenic-based pesticides (in Australia, New Zealand and the US) are the major anthropogenic sources that contribute to arsenic pollution [20,21]. In contrast, selenium exists naturally as a trace element. Selenium pollution is typically associated with the release of selenium-containing waste products from a broad spectrum of anthropogenic activities, ranging from mining (coal, gold, silver and nickel, phosphate), municipal landfills, petrochemical processing (oil transport, refining and utilization), to agricultural irrigation and industrial manufacturing operations [4]. Due to these important practical issues of arsenite and selenite in aquatic environment, we chose these potential metalloid pollutants for a comparison study of biosensors.

## 2. Materials and Methods

### 2.1. Chemicals

Menadione (2-methyl-1,4-naphtaquinone) was obtained from Mitsuwa Chemical Co., Ltd. (Hiratsuka, Japan). Sodium arsenite was a product of Wako Pure Chemicals Industries (Osaka, Japan). Disodium selenite was purchased from Santoku Chemical Industries Co., Ltd. (Tokyo, Japan). All other chemicals were of highest purity available. Chemical stocks were prepared by dissolving in Milli-Q water (Nihon Millipore KK, Tokyo, Japan) or analytical grade DMSO.

### 2.2. Bacterial Strain

The bacterial strain involved in this study was *Escherichia coli* strain DH5α^TM^ (Life Technologies, Tokyo, Japan) transformed with the plasmid pRSET-roGFP2 [3]. Ampicillin (100 μg/mL) was added to both Luria-Bertani agar and liquid medium for the purpose of plasmid maintenance, as previously reported [3].

### 2.3. Fabrication of High-Throughput Oxidative Stress Biosensor

#### 2.3.1. Detection of Cellular Oxidation Using *E. coli* roGFP2 Cells in Liquid Suspension

*E. coli* cells expressing roGFP2 protein (*E. coli* roGFP2) were cultured, harvested, and suspended in 5 mM HEPES buffer (pH 7.0) containing 171 mM NaCl, and left to settle at 20 °C for 1 h as mentioned in [3]. Fluorescence intensity of *E. coli* roGFP2 in buffer suspension was measured with a spectrofluorophotometer (RF-5300PC, Shimadzu Corporation, Kyoto, Japan). The emission wavelength was 525 nm. The excitation wavelength was scanned along 350–510 nm with FAST mode. Excitation and emission band widths were set at 3 and 10 nm, respectively. Fluorescence emission of *E. coli* roGFP2 cells was recorded before and after the addition of menadione at 0.17, 2, 4, 8 and 16 min. *E. coli* roGFP2 suspension in the cuvette was stirred continuously using a magnetic bar to ensure homogeneity. The ratio of the readings at excitation peaks of 400 and 490 nm was measured as fluorescence ratio (Ex_400 nm_/Ex_490 nm_) [3,12].

#### 2.3.2. Optimization of *E. coli* roGFP2 Immobilization

Two parameters were optimized for the fabrication of *E. coli* roGFP2 oxidative stress biosensor: (i) the concentration of *k*-carrageenan (KC), the immobilization matrix; and (ii) the cell density of *E. coli* roGFP2 cells to be immobilized. Step (i) was carried out by immobilizing 50 μL of *E. coli* roGFP2 cells in buffer suspension (maintained at 35–40 °C) in 50 μL KC, maintained at 40–45 °C, with concentrations of 1.0, 1.2, 1.4, 1.6 and 1.8 (w/v) % respectively. Cell suspension and KC solution were mixed with micropipette tip to ensure even distribution of cells and were solidified in wells of a black flat bottom 96-well microplate (BD Falcon, Franklin Lakes, NJ, USA). Microplates with immobilized *E. coli* roGFP2 cells were then sealed with a sheet of parafilm and kept in 4 °C overnight for complete solidification. Step (ii) of optimization was done by fixing the concentration of KC to 1.8 (w/v) % while varying the cell density of *E. coli* roGFP2. *E. coli* roGFP2 cells and KC were mixed in 1:1 ratio and immobilized as mentioned above in step (i). Immobilized *E. coli* roGFP2 oxidative stress biosensors were exposed to 50 μL of test solution. Fluorescence of the oxidative stress biosensor was measured every 1 min for 10 min using fluorescence microplate reader (Powerscan HT, Dainippon Sumitomo Pharma, Osaka, Japan). Emission filter of 528 nm (bandwidth, 20 nm) and excitation filters of 400 nm (bandwidth, 10 nm) and 490 nm (band width, 20 nm) were used as in [3]. The response of the oxidative stress biosensor was determined by the measurement of cellular oxidation index (COI), where:

$$\text{COI}(t)=\left[\frac{Ex400_{\left(t=x\right)}}{Ex490_{\left(t=x\right)}}\right]-\left[\frac{Ex400_{\left(t=0\right)}}{Ex490_{\left(t=0\right)}}\right]$$

### 2.4. Performance Assessment of E. coli roGFP2 Oxidative Stress Biosensor

The *E. coli* roGFP2 oxidative stress biosensor was exposed to two metalloid compounds: sodium arsenite (NaAsO_3_) and sodium selenite (Na_2_SeO_3_). COI was recorded as mentioned in Section 2.3.2. Both chemicals were prepared with 10^−1^ dilution each level starting from 100 mg·L^−1^ to 0.1 μg·L^−1^. The dose-response relationship and limit of detection (LOD) for both metalloids were determined.

## 3. Results

### 3.1. E. coli roGFP2 Cells in the Detection of Oxidative Stress

**Figure 1.**
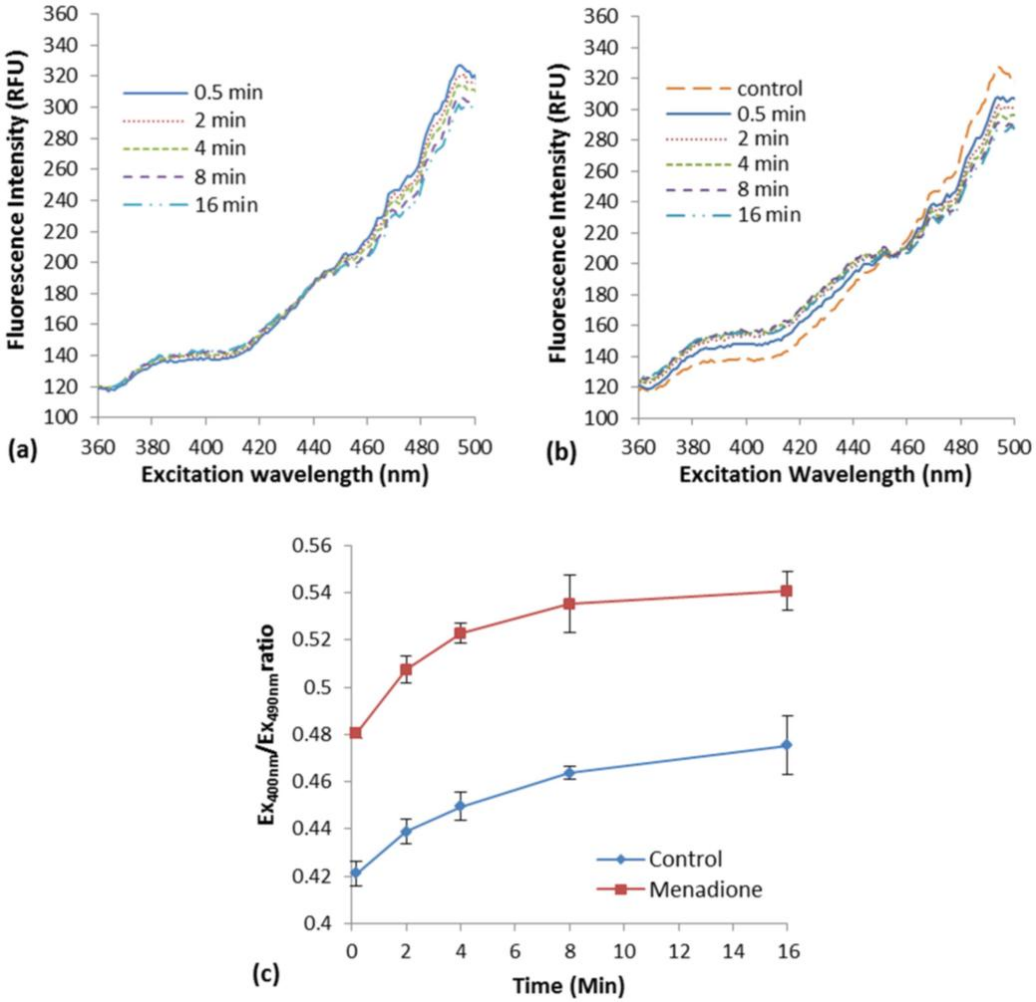
(**a**) Excitation spectra of *E. coli* roGFP2 cells in buffer suspension without menadione; (**b**) Excitation spectra of *E. coli* roGFP2 cells in buffer suspension after exposure to menadione. Spectra are representative ones for each treatment. “Control” in (b) represents 0.5 min excitation spectra of *E. coli* roGFP2 in buffer suspension. RFU, relative fluorescence unit; (**c**) Changes in fluorescence ratio of *E. coli* roGFP2 cells in HEPES buffer suspension, pH 7.0 in the presence of 1 mg·L^−1^ menadione (*n* = 3). Error bars indicate standard deviation.

The induction of oxidative stress in *E. coli* roGFP2 cells with the exposure to menadione in liquid suspension was examined. An increment in excitation spectrum peak at 400 nm and a decrement at 490 nm was observed after menadione exposure (Figure 1b) as compared with the modest response of biosensor in rest (Figure 1a). An apparent rapid increase of fluorescence excited with 400-nm excitation by exposure to 1 mg·L^−1^ menadione was observed, in an opposite fashion with the excitation by 490 nm (Figure 1b). This was a typical oxidation response of roGFP2-expressing *E. coli* cells [3].

A slow cellular oxidation was observed without menadione exposure (Figure 1a). The ratio of fluorescence intensity at peak 400 and 490 nm (COI value) was determined along 16-min of the exposure (Figure 1c). A significant increment of COI was observed immediately after the exposure of *E. coli* roGFP2 cells to menadione (∼10 s). Continuous increase in COI was observed reaching a plateau in 16 min of exposure time. The oxidation kinetics of the control was apparently slower. This indicates that *E. coli* roGFP2 is sensitive in detecting oxidation of cells induced by oxidizing agent, and is suitable to be used in the fabrication of an oxidative stress biosensor.

### 3.2. Fabrication and Optimization of E. coli roGFP2 Oxidative Stress Biosensor

Optimal conditions of the immobilization matrix and cell density were determined to prepare an immobilized biosensor that is more stable and sensitive than a suspension biosensor. *k*-carrageenan (KC) gel matrix was selected because it is comparatively non-toxic to bacterial cells, it forms rigid transparent gel which allows fluorescence detection and its gelling temperature is tolerable by bacterial cells.

#### 3.2.1. Effect of Immobilization Matrix Concentration on the Response of the Biosensor

**Figure 2.**
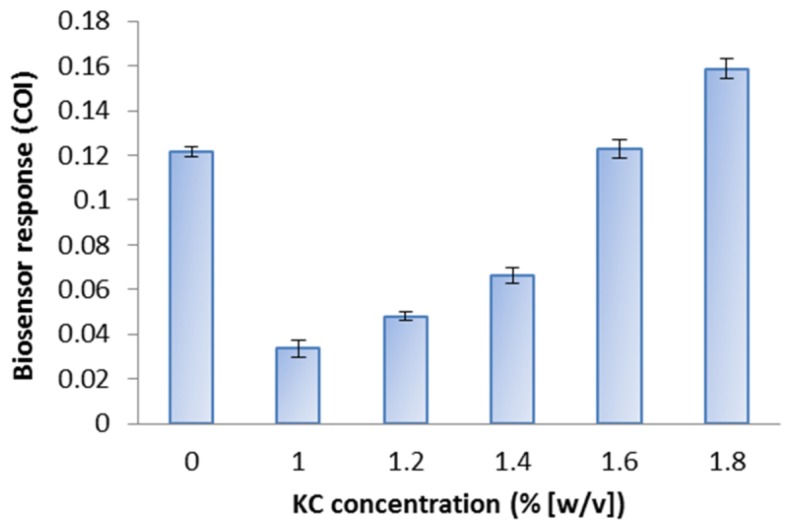
Responses of *E. coli* roGFP2 oxidative stress biosensors embed in several concentrations of KC. The cell density of *E. coli* roGFP2 was fixed at 4.5 × 10^8^ cfu·mL^−1^. The biosensor was exposed to 1 mg·L^−1^ menadione. Cells suspended in the HEPES buffer act as control (0%). Error bars indicate standard deviation (*n* = 8).

The highest COI against 1 mg·L^−1^ menadione exposure was obtained at 1.8% (w/v) KC (Figure 2). In 1.0% to 1.4% (w/v) KC gel, the COI was lower than suspended cells in the buffer (0% KC). Increasing sensitivity was observed for biosensor prepared in increasing KC concentration. Higher concentrations (greater than or equal to 2% w/v) of KC were not suitable for a practical use at the temperature that keeps *E. coli* cells vital, due to the very rapid gelation.

#### 3.2.2. Effect of Cell Density of Immobilized Cells

**Figure 3.**
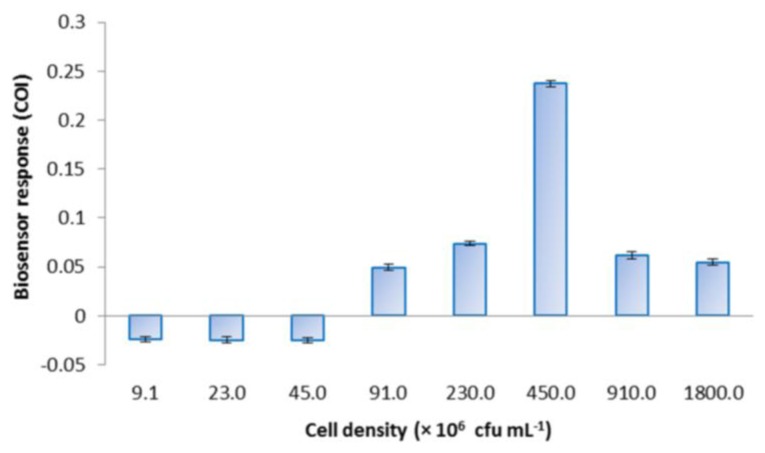
Responses of *E. coli* roGFP2 oxidative stress biosensors prepared with various cell densities immobilized in 1.8% (w/v) KC to 1 mg·L^−1^ menadione exposure. Error bars indicate the values of standard deviation (*n* = 8). Negative COI values indicate apparent reduction of the biosensors.

*E. coli* roGFP2 oxidative stress biosensor was prepared with different cell densities and challenged with 1 mg·L^−1^ menadione to determine the optimal density for the biosensor response (Figure 3). COI increased following the increase in cell density from 4.5 × 10^6^ to 4.5 × 10^8^ cfu·mL^−1^. A maximal response was observed at 4.5 × 10^8^ cfu·mL^−1^. Cell densities exceeding the optimum (810 and 1800 × 10^6^ cfu·mL^−1^) resulted in decreased COI.

### 3.3. Application of E. coli roGFP2 Oxidative Stress Biosensor

#### 3.3.1. Detection of Arsenite with *E. coli* roGFP2 Oxidative Stress Biosensor

**Figure 4.**
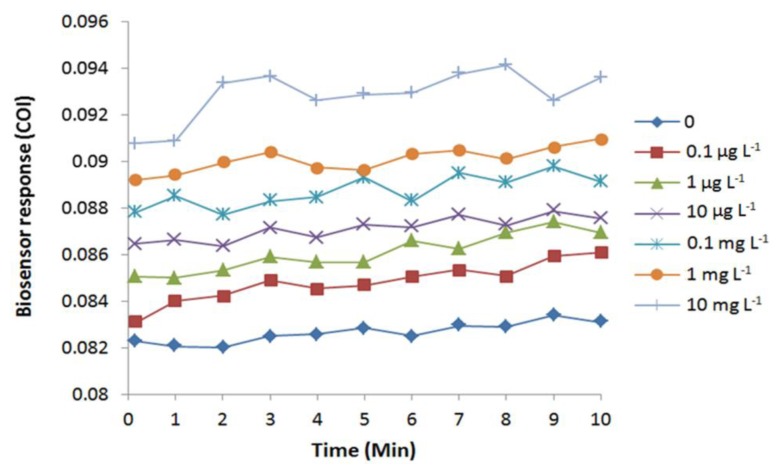
Cellular oxidation profile of *E. coli* roGFP2 oxidative stress biosensor exposed to NaAsO_3_ (0.1 μg·L^−1^–100 mg·L^−1^). A typical result out of 4 replicates is shown. The roGFP2-expressing *E. coli* cells were immobilized in 1.8% (w/v) KC at 4.5 × 10^8^ cfu·mL^−1^.

Exposure to arsenite induced oxidative stress in *E. coli* cells, as predicted by an immediate increase of COI (Figure 4). The increase in COI was observed immediately after the arsenite exposure. Due to mechanical lag, the reading of fluorescence began 10 s after the application of the chemical. Therefore, the biosensor detected cellular oxidative stress faster or equal to 10 s by an exposure to 0.1 μg·L^−1^ or higher concentrations of arsenite. Completion of arsenite-induced cellular oxidation was observed at 3 to 9 min depending on the tested concentrations.

**Figure 5.**
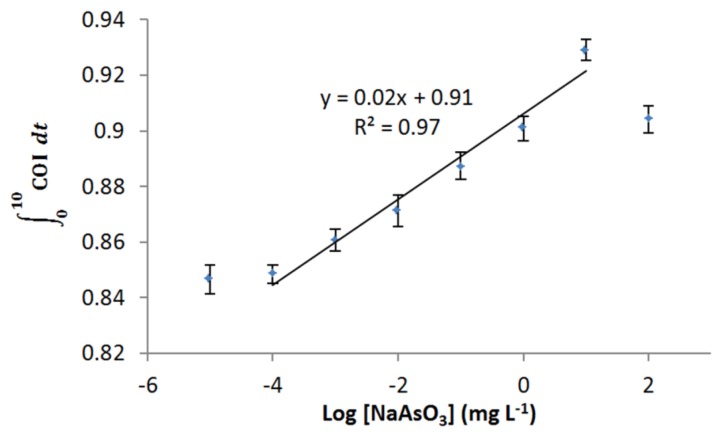
Dynamic range of *E. coli* roGFP2 oxidative stress biosensor towards NaAsO_3_ exposure (10 ng·L^−1^–100 mg·L^−1^). $\int_{0}^{10}\text{COI}\;dt$ indicates the total COI response of biosensor in 10 min. Error bars indicate the values of standard deviation (*n* = 8). Solid line and inset equation indicate linear regression of the plots. The roGFP2-expressing *E. coli* cells were immobilized in 1.8% (w/v) KC at 4.5 × 10^8^ cfu·mL^−1^.

Dose-dependent response of *E. coli* roGFP2 oxidative stress biosensor to arsenite was examined (Figure 5). The biosensor showed a wide linear detection range for arsenite ranging from 1 μg·L^−1^ to 10 mg·L^−1^. Limit of detection (LOD) for arsenite-induced oxidative stress was determined as 0.2 μg·L^−1^ by an extrapolation. No increment in COI was detected for the exposure to 10 ng·L^−1^ arsenite. These characteristics indicate that the *E. coli* roGFP2 biosensor is an extremely rapid sensitive sensor to detect oxidative cellular damage caused by arsenite.

#### 3.3.2. Detection of Selenite with *E. coli* roGFP2 Oxidative Stress Biosensor

**Figure 6.**
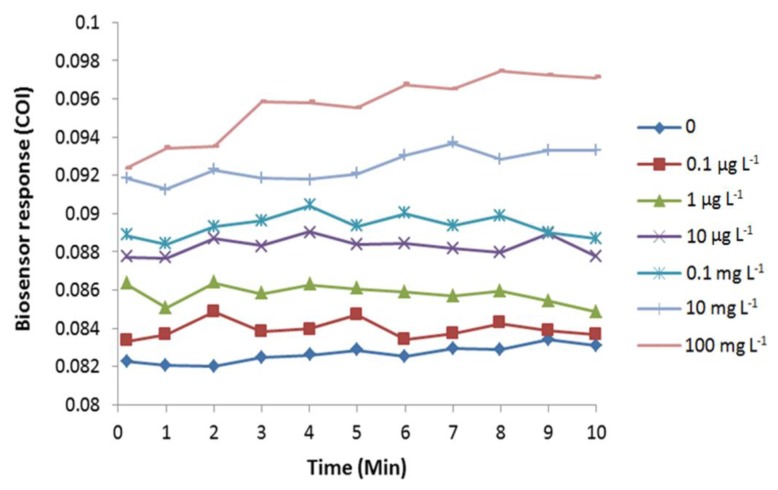
Cellular oxidation profile of *E. coli* roGFP2 oxidative stress biosensor exposed to Na_2_SeO_3_ (0.1 μg·L^−1^–100 mg·L^−1^). A typical result out of 4 replicates is shown. The roGFP2-expressing *E. coli* cells were immobilized in 1.8% (w/v) KC at 4.5 × 10^8^ cfu·mL^−1^.

**Figure 7.**
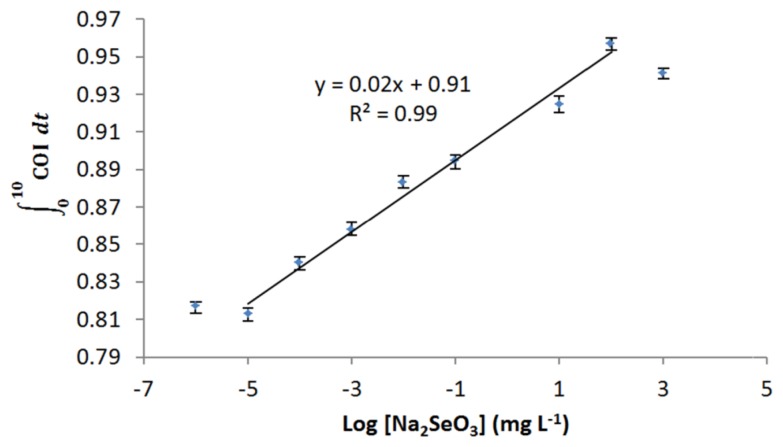
Dynamic range of *E. coli* roGFP2 oxidative stress biosensor towards the exposure of Na_2_SeO_3_ (1 ng·L^−1^–1000 mg·L^−1^). $\int_{0}^{10}\text{COI}\;dt$ indicates the total COI response of biosensor in 10 min. Error bars indicate the values of standard deviation (*n* = 8). The roGFP2-expressing *E. coli* cells were immobilized in 1.8% (w/v) KC at 4.5 × 10^8^ cfu·mL^−1^.

Selenite induced increase of COI in a time dependent manner, suggesting *E. coli* cells were oxidized by the selenite exposure (Figure 6). Similar to the response to arsenite, cellular oxidation occurred immediately after the exposure (faster or equal to 10 s). Response achieved full cellular oxidation to selenite exposure at 8 min, except for 0.1 mg·L^−1^. The oxidation response was apparently dose-dependent. Linear response was observed from 10 ng·L^−1^ to 100 mg·L^−1^ (Figure 7), showing a very wide dynamic range. LOD was determined as low as 5.8 ng·L^−1^.

## 4. Discussion

In this study, a sensitive oxidative stress biosensor made up of immobilized *E. coli* roGFP2 cells was fabricated and its performance in detecting metalloid-inducing oxidation was assessed. A novel and simple immobilization procedure was developed to stably fix the *E. coli* roGFP2 cells with a non-toxic plant-based KC matrix. Ratiometric fluorescence measurement enables close to real time monitor as compared to conventional oxidative stress monitoring which took up to hours or days [6]. *E. coli* roGFP2 forms two fluorescence excitation peaks at around 400 and 490 nm which show reversible changes in ratio following cellular redox potential fluctuating by cellular stresses (Figure 1).

### 4.1. Optimization of E. coli roGFP2 Immobilization

*E. coli* roGFP2 cells were immobilized in KC matrix to achieve improved biosensor properties. KC concentration and bacterial cell density optimizations were studied (Figures 2 and 3). KC concentrations below 1.0% w/v and above 1.8% (w/v) were not studied as the gel appeared to be too watery at lower concentrations and found to be too sticky to achieve homogeneity at higher concentrations. The lower extent of biosensor response for *E. coli* roGFP2 immobilized in 1.0%–1.6% (w/v) KC can be explained by the stacking of bacterial cells. Weaker KC bonding occurs in KC gel at lower concentrations, which may not stably hold the *E. coli* roGFP2 cells, allowing cells to accumulate on the bottom of the well. Fluorescence signals could not be recorded accurately when the cells were not distributed evenly in the gel. This explains the results of the biosensor prepared in 1.0% (w/v) gel which gave a lower COI value than the cells suspended in buffer. This was due to increasing interaction of KC polymer chains with increasing concentration [22]. KC double helix aggregation with the presence of Na^+^ cation from the suspension buffer decreases electrostatic repulsion between the sulphate groups resulting in the formation of a double helix domain which contributes to rigid KC matrix on higher concentration [23–25]. With 1.8% (w/v) KC as the optimal matrix concentration, 4.5 × 10^8^ cfu·mL^−1^ appeared to be the optimum cell density. Positive COI values indicated the detection of oxidative stress by the biosensors. This study provides essential information for possible application of the *E. coli* roGFP2 biosensor in practice.

### 4.2. Lifetime of the E. coli roGFP2 Oxidative Stress Biosensor

The stability of the optimized *E. coli* roGFP2 oxidative stress biosensor was studied for a period of 49 days in our previous study [26]. Long term stability study of the biosensor showed encouraging results. A stable lifespan of 46 days was observed for the *E. coli* roGFP2 oxidative stress biosensor, with fluorescence intensity of 442.04 ± 13.94 a.u. in the first 43 days, a small decrease (19%–23%) on the 44–46th day and a drastic 79.36% drop detected on the 47th day. A decrease to 2.8% of the initial readings was observed two days later. We thus concluded that the biosensor was viable for 46 days of storage at 4 °C. The decrease in fluorescence intensity later was caused by cell death, either due to the lack of nutrients or toxicity effects of the bacterial respiratory wastes. Immobilization of cells in KC for the purpose of fabrication of whole-cell biosensor has not been a popular approach. KC is a non-toxic gelation fiber and is industrially available [23,25]. These properties are favorable characteristics for use as an immobilization matrix of whole-cell biosensors [27]. Immobilization via matrix entrapment is commonly done using gelatin, agarose, polyacrylamide, cellulose acetate *etc.*, [9,28,29]. Compared to KC, gelatin has a lower melting point (∼30 °C), which is not optimum for the activity of *E. coli* cells. Agarose is rather stiff and brittle compared with KC. Most reported biosensors using entrapment matrixes have short lifetimes, high probability of leakage and lower sensitivity [9,30]. This study is an example showing that KC is suitable for the fabrication of biosensors.

### 4.3. Limit of Dynamic Range

Arsenic is well-known for its toxicity, with trivalent arsenite being a more highly bioreactive form than pentavalent arsenate [2,31], as trivalent arsenite is taken up faster by the cells compared to pentavalent species. The toxic effect of arsenite causing *E. coli* roGFP2 oxidative stress is estimated to be related with the formation of OH• radicals via the Haber Weiss reaction in the GSH pathway in the cell [2,5]. OH• reacts with cysteine groups on roGFP2 protein ends with the formation of superoxide ion, which will activate the GSH pathway. The high affinity of arsenite towards GSH decreases the GSH level, while enhances the ocurrence of oxidative stress in *E. coli* roGFP2, which thereafter leads to changes of the roGFP2 chromophore's conformation that gives the detected response. The antioxidant system in *E. coli* roGFP2 is able to stabilize the instability of redox stage in the cell before oxidative stress ocurrs. Therefore, LOD against arsenic is attributed to the capacity of the reduction-oxidation buffering pool in cells. Cellular oxidation of the biosensor dropped with 100 mg·L^−1^ arsenite exposure due to the cytotoxic effect of arsenite towards the bacterial cells, where *E. coli* roGFP2 cells might be killed and roGFP2 might be biochemically damaged directly (Figures 4 and 5). The upper limit of the dynamic range of biosensor response may be attained by dysfunction of roGFP2 in cells.

Selenium compounds share the same mode of action in oxidation of cells, while there is difference in the oxidation states (As[V], As[III], As[0], As[-III], Se[VI], Se[IV], Se[0], and Se[-II]). Both selenium and arsenic are essential elements for *E. coli* where they are needed as a trace amount for growth, metabolism and life cycle. However, they become toxic at high concentrations [32]. Few studies on selenite toxicity have been carried out. Nevertheless, studies have shown that reduction of selenite with GSH and thiol groups in cells enhances the formation of superoxide and H_2_O_2_ [33,34]. Selenite toxicity in *E. coli* roGFP2 becomes apparent when the level of oxidative stress exceeds the cellular antioxidant defenses of the cell (Figures 6 and 7). The *E. coli* roGFP2 oxidative stress biosensor shows a concentration-dependent response to both arsenite- and selenite-induced cellular oxidations with different levels of sensitivity. The LOD for the biosensor in selenite oxidation is ∼34.5 times lower as compared with arsenite. Selenite may have an effect on the reduction-oxidation buffering pool at lower concentration. The highest point in the linear range for selenite-induced oxidative stress is also higher as compared with arsenite, due to the higher toxicity of arsenite [3]. It is therefore most likely that roGFP2 was not damaged by selenite as much as arsenite.

### 4.4. Comparison with Reported Biosensors

The fabricated *E. coli* roGFP2 oxidative stress biosensor was compared with previously reported arsenite and selenite detection biosensors. It was to our surprise that only a few whole-cell biosensors were reported to be fabricated for this purpose. To our best knowledge, this study is the first report of an immobilized whole-cell biosensor for detecting metalloids. The metalloid biosensors reported by Arias-Barreiro *et al.*, Fujimoto *et al.* and Dwivedi *et al.* were based on free-moving bacterial cells suspended in either buffer or culture medium [3,35,36]. The oxidative stress biosensor reported by [3] was based on the same biochemistry mechanism reported in this paper, while for [35] and [36], the biosensor responses were based on a colour change of a bacterial pigment or biosensor medium due to enzyme transformations or changes in a bioreduction compound, respectively. A summary of the properties of four whole-cell biosensors is shown in Table 1.

Our *E. coli* roGFP2 oxidative stress biosensor has a wider linear range as compared with the previously reported arsenite and selenite biosensors. The LODs of the oxidative stress biosensor reported in this study appear to be the lowest among all, except for Arias-Barreiro *et al.* [3] that was based on the same detection principle. Both of the reported *E. coli* roGFP2 biosensors in the table have short response time. Our biosensor took an additional of 2 min to reach a plateau, because time was needed for the analytes to diffuse through the immobilization matrix. The *E. coli* roGFP2 oxidative stress biosensor also reported to be stable for long term storage and with high percentage of reproducibility.

### 4.5. Anticipated Advantages of the E. coli roGFP2 Biosensor

The response time of the KC-entrapped biosensor was quick, and comparable to the free-moving cell suspension biosensor in the previous study [3], despite the immobilization. The biosensor is analyzed in 96-well microplate format. These characteristics allow high throughput whole effluent toxicity testing [37]. Compared to conventional toxicity bioassays, only a small amount of water sample is needed for the biosensor (50 μL for the biosensor; a few milliliters to several liters for Microtox test, crustacean immobilization test and fish mortality test) [38–40]. This offers a great advantage in toxicity identification and evaluation procedures [41–43], which require several treatments, fractionation and repeated assays.

The biosensors detect the degree of oxidative stress burdened on cells. Thus, the biosensor response should reflect the joint action of toxicants within the limits of toxic mode of action through cellular oxidation. With the criteria of quick response, long-term stability, high reproducibility and high sensitivity (Table 1), our biosensor has the potential to be applied as an early warning system in toxicity assessment, environmental monitoring and future environmental risk management approaches, achieving integrated watershed management based on cytotoxicity.

## 5. Conclusions

A novel sensitive *E. coli* roGFP2 oxidative stress biosensor was fabricated for the detection of oxidative stress-inducing chemicals. The biosensor was able to detect cellular oxidation inducing metalloids up to μg·L^−1^ and ng·L^−1^ levels with a wide dynamic range. Combined with a multiplate reader, which was able to read the response of the biosensor immobilized in a 96-well plate in as short a time as 10 s, a quick sensing high-throughput sensing array was made possible. This research was able to illustrate future development of a high-throughput, real-time environmental toxicity monitoring system which is time-saving, sensitive and has long term stability.

## Figures and Tables

**Table 1.** Performance comparison of *E. coli* roGFP2 oxidative stress biosensor with reported whole-cell oxidative stress biosensors using arsenite and/or selenite as the analyte(s).

**Parameter**	**This Study**	**Arias-Barreiro *et al.* 2010 [3]**	**Fujimoto *et al.* 2006 [35]**	**Dwivedi *et al.* 2013 [36]**
Bacteria	*E. coli* DH5α expressing *roGFP2*	*E. coli* DH5α expressing *roGFP2*	*Rhodovulum sulfidophilum* CDM2	*Pseudomonas aeruginosa* JS-11

Chemicals tested	Arsenite and selenite	Heavy metals, metalloids, pesticides *etc.*	Arsenite	Selenite

Reproducibility (%RSD)	2.03	-	-	-

Linear range (mg·L^−1^)	Arsenite: 1.0 × 10^−3^–1.0 × 10^1^	Arsenite: 2.89 × 10^−6^–7.14 × 10^−3^	2.0 × 10^−3^–1.0 × 10^−2^	0–4.32
	Selenite: 1.0 × 10^−5^–1.0 × 10^2^	Selenite: 3.95–89.8		

LOD (mg·L^−1^)	Arsenite: 2.0 × 10^−4^	Arsenite: 1.0 × 10^−7^	3.0 × 10^−3^	EC50 = 23.21
	Selenite: 5.8 × 10^−6^	Selenite: 3.1		

Response time (min)	0–9	0–7	180–1440	2880

Stability (days)	46	∼0.42	-	-
